# Electrocardiogram Changes of Donepezil Administration in Elderly Patients with Ischemic Heart Disease

**DOI:** 10.1155/2018/9141320

**Published:** 2018-04-23

**Authors:** Deguo Wang, Yong Wu, Ancai Wang, Yueyun Chen, Ting Zhang, Nengwei Hu

**Affiliations:** ^1^Department of Gerontology, Yijishan Hospital of Wannan Medical College, Wuhu, Anhui 241001, China; ^**2**^ Department of Psychology, Wannan Medical College, Wuhu, Anhui 241001, China; ^3^Department of Pharmacology and Therapeutics, Institute of Neuroscience, Trinity College Dublin, Dublin, Ireland

## Abstract

**Objective:**

Donepezil, a widely used cholinesterase inhibitor for treating Alzheimer's disease, has been reported to induce bradyarrhythmias and torsade de pointes. In this study, we aimed at determining electrocardiogram changes of donepezil administration in elderly patients with ischemic heart disease, who tend to suffer from cognitive disorders.

**Methods:**

Sixty patients with ischemic heart disease and mild cognitive impairment were treated with donepezil (5 mg/day) and followed up for at least four weeks. A twenty-four-hour ambulatory electrocardiogram was performed for the analysis of heart rate variability. The ECG parameters including heart rate (HR), PR and RR intervals, QT interval, and QRS duration were recorded at the baseline and after donepezil administration.

**Results:**

Donepezil administration resulted in significant reduction in mean HR and the lowest HR and prolongation of PR and RR intervals, whereas it had no significant effects on QRS duration and QT parameters including QT, corrected QT interval, QT dispersion, and T_peak-end_ interval. HRV analysis showed that donepezil administration significantly improved parasympathetic function, indicated by decreased low/high frequency (LF/HF) ratio and high frequency (HF) components and oscillation of RR intervals.

**Conclusions:**

These data demonstrated that donepezil administration decreased HR, prolonged PR interval, and increased parasympathetic function without affecting QRS duration and QT intervals, suggesting that it can be used safely in elderly patients with ischemic heart disease.

## 1. Introduction

Cardiovascular disease accounts for one-third of the deaths worldwide, particularly in aging population [1]. Some of them suffer from cognitive impairment. Therefore, combination therapy with cardiovascular and psychiatric drugs is very common [2]. Cholinesterase inhibitors are currently prescribed as the first line therapy for Alzheimer's disease (AD) and cognitive impairment [3, 4]. By inhibiting the enzyme acetylcholinesterase, cholinesterase inhibitors reduce breakdown of acetylcholine, which is associated with the improvement in memory function. As these drugs are continuously used, their side effects should be treated. Until now, the most of unwanted effects are involved mainly in the gastrointestinal system, which can be well tolerated [3, 5].

Theoretically, cholinesterase inhibitors can cause some cardiovascular effects, including bradycardia, conductive block, and QT prolongation [6, 7], because the heart is rich in cholinesterase. Several case reports showed that donepezil could lead rarely to serious bradycardia needing pacemaker implantation and fatal ventricular arrhythmia (torsade de pointes (TdP)) [7, 8]. However, the vagotonic effect of cholinesterase inhibitors might also protect the heart against ischemia and dysarrhythmias. Experimental studies suggested that donepezil improved the long-term survival of congestive heart failure rats by preventing pump failure and cardiac remodeling and attenuating atherogenesis [9, 10]. Interestingly, a population-based study showed that donepezil actually significantly reduced risk of cardiac pacemaker insertion [11]. Another nationwide cohort study revealed that donepezil reduced the risk of myocardial infarction and death in subjects with AD [12]. In the present study, we examined the effects of donepezil on ECG parameters including heart rate (HR), QT interval, corrected QT interval (QTc), QT interval dispersion (QTd), and T_peak-end_ interval, which are associated with the risk of bradycardia and TdP.

Higher sympathetic and lower parasympathetic functions were found to be associated with lower cognitive performance in patients with AD [13] and all-cause mortality and arrhythmic death in patients with ischemic heart disease [14]. In the present study, we examined cardiac autonomic function using heart rate variability (HRV) analysis. HRV is the beat-to-beat oscillation of RR intervals in an electrocardiogram. Until now, it is a reliable tool for evaluating autonomic nervous system activities by characterizing the relative and absolute parasympathetic and sympathetic modulations and the sympathovagal balance of the heart [15].

In the present study, we focused on ischemic heart disease patients complicated with mild cognitive impairment (MCI). By analyzing HRV and electrocardiogram parameters, we determined whether donepezil is associated with an increased risk of bradycardia and ventricular arrhythmia.

## 2. Methods

### 2.1. Study Subjects

The study included elderly patients (>65 years) with established coronary artery disease with mild cognitive impairment, who visited our hospital between January 1, 2015, to September 31, 2017. Patients would be selected to the study if they had not been described with any cholinesterase inhibitors within 3 months according to the previous report [7]. Ischemic heart disease was diagnosed, including pervious myocardial infarction, stable angina pectoris, abnormal cardiac exercise test , or plaque by prior imaging in at least one coronary artery. The abnormal cardiac exercise test results including horizontal or down sloping ST depression, J-point elevation, angina pectoris, and blood pressure decline confirm the presence of ischemic heart disease [16].

Mild cognitive impairment (MCI) was diagnosed by a psychiatrist according to Chinese guidelines for diagnosis and management of cognitive impairment and dementia [17]. Mini-Mental State Examination (MMSE) [18] and Alzheimer's Disease Cooperative Study Scale for activities of daily life in MCI (ADCS-MCI-ADL) were used as a brief screening tool for MCI. The exclusion criteria were patients with atrial fibrillation, acute myocardial infarction, or acute coronary syndrome in the past 3 months; patients with bradycardia; and patients with artificial cardiac pacing or using antiarrhythmic agents.

### 2.2. Study Procedures

Before participation, all patients gave written informed consent to participate in the study, which was approved by the Institutional Research Ethics Committee, in accordance with Chinese guidelines for ethical review of drug clinical trials. After careful history taking, examination and laboratory evaluation (plasma glucose, potassium, magnesium and calcium), electrocardiogram (ECG), and Holter ECG measurement, the selected patients began to take donepezil once a day (5 mg) continuously for at least four weeks. During the 4-week follow-up, if the patients complained of syncope, palpitation, fatigue, or weakness, they were recalled to hospital and ECG measurement was taken immediately. Because QTc tends to respond to some drugs and electrolytes [15], the readmission was postponed if subjects had to take these drugs additionally or had electrolyte disturbances. Once the follow-up was completed in the subjects, ECG and Holter ECG was measured immediately.

### 2.3. Electrocardiogram Measurement

All patients were recorded a 12-lead surface ECG measurement by using 25 mm/second paper speed and standardized at 0.1 mV/mm after the patients had rested for at least 10 minutes in the supine position. ECG parameters, including HR, PR, QT, QTc, QTd, and T peak-to-end (T_peak-end_) interval, and QRS duration were calculated by two examiners blinded to the clinical characteristics of patients and groups [19]. The QT interval was corrected for the heart rate by using Bazett's formula (QTc = QT/square root of RR). QTd is defined as the difference between the maximum and minimum QT interval measured on the 12-lead. T_peak-end_ interval was measured in lead V5 from the peak of the T wave to the end of the T wave, defined as the point of return to the T-P baseline. If U waves were present, we defined the end of the T wave as the nadir of the curve between the T and U waves.

### 2.4. Holter ECG and HRV Measurement

A 12-leads Holter electrocardiogram (DMS300-4, Diagnostic Monitoring Software, NV, USA) was performed in the participants for 24 hours. Because bradycardia is a relatively common occurrence in older patients after using cholinesterase inhibitors [6, 7], Holter ECG parameters such as mean heart rate (HR), the lowest HR, and the longest RR were determined.

HRV measurement was carried out before and four weeks after donepezil administration. The electrocardiogram data were analyzed off-line for HRV using DMS (Diagnostic Monitoring Software, USA). The resultant tachogram underwent 512 point fast Fourier transform with a Hanning window to calculate the power spectrum of HRV, with the low frequency (LF) component at 0.040–0.150 Hz and the high frequency (HF) component at 0.150–0.401 Hz. A typical spectrum of RR series is characterized by two main components: the LF component and the HF component. The HF component reflects cardiac vagal tone, whereas the LF component reflects both sympathetic and parasympathetic modulation of the heart rate. The LF/HF ratio is an index of sympathetic activity [15].

### 2.5. Statistical Analysis

Data were presented as the number (*n*) for nominal variables and as the mean ± SD for continuous variables. The distribution characteristics of the variables were evaluated by the Kolmogorov–Smirnov test. If the variables were of normal distribution, comparisons were tested by Student's *t*-test. Otherwise, comparisons were tested for statistical significance by using the Mann–Whitney *U* test for nonnormal distribution data. Statistical analyses were performed using the SPSS software, version 16.0. The differences were considered to be significant at *P* < 0.05.

## 3. Results

A total of 60 elderly patients were enrolled and completed the study including 37 male and 23 female subjects. The demographics and baseline characteristics of the patients are shown in Table 1. Blood pressure and heart rate of all individuals prior to donepezil were normal. The values of plasma glucose and plasma ions such as potassium, magnesium, and calcium were within normal range. The average follow-up period was 35.1 ± 4.6 days. During the period of follow-up, no any donepezil-related adverse effects were reported such as syncope, palpitation, fatigue, and weakness. Moreover, there were no bradycardia events leading to rehospitalization.

As shown in Table 2, mean HR and the lowest HR significantly reduced after donepezil administration compared with baseline (*P* < 0.05). The longest RR interval was significantly prolonged with respect to baseline (*P* < 0.05). There was no significant difference in LF with or without donepezil administration. HF increased significantly after donepezil intervention. As a result, the LF/HF ratio decreased significantly after donepezil administration.

As shown in Table 3, the mean heart rate significantly reduced after donepezil intervention (*P* < 0.05). The mean PR interval was significantly prolonged after donepezil administration (*P* < 0.05). Moreover, the QRS duration and QT parameters including QT, QTc, QTd, and T_peak-end_ interval did not change significantly as a result of donepezil treatment and discontinuation.

## 4. Discussions

In the present study, we observed the cardiac effects of donepezil administration in patients with ischemic heart disease and focused on the risk of bradycardia and ventricular arrhythmia. Our data revealed two findings: (1) donepezil increased cardiac vagal tone and reduced cardiac sympathetic activity; (2) donepezil prolonged RR and PR interval without changing QRS duration and QT intervals.

Imbalance of the autonomic nervous system with higher sympathetic and lower parasympathetic function occurs in both AD and ischemic heart disease [13, 14]. Treatment with acetylcholinesterase inhibitors may lead to bradycardia by potentiating cardiac vagal activity [20]. Previous studies suggested that treatment for AD with acetylcholinesterase inhibitors might result in bradyarrhythmias even needing pacemaker implantation [6, 7, 21]. However, these studies defined bradycardia as heart rate lower than 60 beats/minute. Most subjects had no symptomatic response to the slow heart rate. Among them, in only a few serious cases, artificial pacemakers had been implanted [7, 11, 21]. In the present study, we founded that donepezil decreased mean HR and the lowest HR and prolonged the longest RR interval. However, we did not observe serious bradycardia needing donepezil retraction or pacemaker implantation.

Because overexcited sympathetic function plays an important role in ischemic heart disease, the vagotonic stimulation of administrated acetylcholinesterase inhibitors might also provide beneficial effects [22]. Actually, recent studies provided strong evidences that donepezil reduces the risk of myocardial infarction and pacemaker implantation in subjects with AD [11, 11, 12]. In two retrospective cohort studies on dementia patients which involved in East Asian, acetylcholinesterase inhibitors are associated with a decreased risk of an acute coronary syndrome event and ischemic stroke [23, 24]. These cardiac benefits of acetylcholinesterase inhibitors might be associated with autonomic modulation against heart diseases. In the present study, our data showed that donepezil administration significantly increased HF and decreased LF/HF ratio, suggesting a shift in autonomic modulation from sympathetic to vagal tone.

Torsade de pointes (TdP) is a fatal ventricular arrhythmia and is associated with prolongation of QT interval which reflects enhanced repolarization dispersion in the myocardium [25]. QT prolongations are thought to be a marker of the fatal ventricular arrhythmia and related to multiple factors, such as aging, hypopotassium, taking antiarrhythmic drugs, and ischemic heart disease [26, 27]. T_peak-end_ has been proposed to transmural dispersion of repolarization, which is related to the vulnerability of ventricular arrhythmias [28]. Prolongation of T_peak-end_ interval has been shown to be associated with ventricular arrhythmias in various cardiac disorders including long QT syndrome [29] and ischemic heart disease [30]. It is documented in a few case reports that donepezil prolonged QT interval and caused TdP [8, 27]. These results could not reflect the pervasive impact of donepezil intervention because some cases had specific disease backgrounds such as unknown sinus sick syndrome, paroxysmal atrial fibrillation, myocardial infarction, or combination therapy with negative chronotropic drugs [27, 31]. On the contrary, Isik and Igeta [32, 33] reported that donepezil administration did not change QT and QTc interval in patients with AD compared with baseline. In the present study, no significant difference in QT, QTc, QTd, and T_peak-end_ interval was detected after 4 weeks of donepezil intervention. These findings suggested that donepezil had no such remarkable effect on ventricular repolarization even in patients with stable coronary artery disease as documented in previous case reports.

## 5. Limitations

Several limitations of the present study should be stated. First, it is a small sample longitudinal study which included only a small number of subjects. Second, we could not deduce the long-term effects of electrocardiogram and HRV because we observed only the effects of donepezil administration for about one month. During such a short period, no any donepezil-related side effects such as bradycardia, conductive block, and QT prolongation had been reported as previously documented [7, 34]. Third, the selected subjects in this study were mainly patients with ischemic heart disease and mild cognitive impairment. Some of them may have other complicated diseases such as hypertension, diabetes mellitus, and cerebral vascular disease. Therefore, it is difficult to analyze electrocardiogram changes in different complicated diseases. Further prospective studies are needed in the future including more subjects and longer time to determine the electrocardiogram changes of donepezil administration.

## 6. Conclusions

Although there are several limitations in this study, it is a novel study focused on patients with ischemic heart disease to determine the effects of donepezil intervention on the risk of bradyarrhythmias and ventricular arrhythmia. This study demonstrated that donepezil causes mild decrease in heart rate and prolongation of PR interval without affecting QRS duration and QT intervals, suggesting that it can be used safely in elderly patients with a history of ischemic heart disease.

## Figures and Tables

**Table 1:** Demographics and baseline characteristics of patients.

Age (year)	75.7 ± 5.8
Gender (male/female)	37/23
Follow-up period (days)	35.1 ± 4.6
SBP (mm·Hg)	136.5 ± 12.3
DBP (mm·Hg)	71.3 ± 9.8
MMSE	21.7 ± 5.1
ADCS-MCI-ADL	25.2 ± 10.2
HR (beats/min)	76.8 ± 10.8
LVEF (%)	67.4 ± 5.8
Glucose (mmol/L)	6.25 ± 1.13
Potassium (mmol/L)	3.94 ± 0.49
Magnesium (mmol/L)	0.93 ± 0.12
Calcium (mmol/L)	2.12 ± 0.13

*Note.* Values are shown as mean ± SD. SBP: systolic blood pressure; DBP: diastolic blood pressure; MMSE: Mini-Mental State Examination; ADCS-MCI-ADL: Alzheimer's Disease Cooperative Study Scale for activities of daily living in MCI; LVEF: left ventricular ejection fraction.

**Table 2:** Holter electrocardiogram and HRV at baseline and 4 weeks after donepezil intervention.

	Baseline	Donepezil	*t*	*p*
Mean HR (beats/min)	74.5 ± 11.3	68.1 ± 10.4	3.2260	0.0013
The lowest HR (beats/min)	53.2 ± 6.4	50.3 ± 6.8	2.4056	0.0177
Longest RR interval(s)	1.36 ± 0.77	1.47 ± 0.84	0.7477	0.4561
LF (ms^2^)	8.7 ± 8.5	8.9 ± 7.7	0.1351	0.8928
HF (ms^2^)	4.6 ± 5.3	6.8 ± 4.6	2.4283	0.0152
LF/HF ratio	2.14 ± 1.12	1.66 ± 1.08	2.3897	0.0169

*Note.* Values are shown as mean ± SD. HR, heart rate; LF, low frequency; HF, high frequency.

**Table 3:** ECG parameters at baseline and 4 weeks after donepezil intervention.

	Baseline	Donepezil	*t*	*p*
HR (beats/min)	76.8 ± 10.8	72.1 ± 12.7^∗^	2.1838	0.0310
PR interval (ms)	172.3 ± 21.3	182.6 ± 25.8^∗^	2.3847	0.0187
QRS duration (ms)	98.2 ± 15.3	96.6 ± 24.3	0.5918	0.5551
QT interval (ms)	378.3 ± 31.3	383.8 ± 32.4	0.9457	0.3462
QTc (ms)	415.1 ± 35.7	424.3 ± 37.2	1.0858	0.7529
QTd (ms)	64.5 ± 10.7	67.2 ± 11.3	1.4082	0.1591
T_peak-end_ (ms)	75.3 ± 12.7	77.3 ± 14.3	0.8100	0.4179

*Note.* Values are shown as mean ± SD. HR, heart rate; QRS, QRS complexes; QTc, corrected QT intervals; QTd, QT dispersion; T_peak-end_, T peak-to-end.
